# To Explore the Predictive Power of Visuomotor Network Dysfunctions in Mild Cognitive Impairment and Alzheimer’s Disease

**DOI:** 10.3389/fnins.2021.654003

**Published:** 2021-06-28

**Authors:** Justine Staal, Francesco Mattace-Raso, Hennie A. M. Daniels, Johannes van der Steen, Johan J. M. Pel

**Affiliations:** ^1^Vestibular and Ocular Motor Research Group, Department of Neuroscience, Erasmus MC, Rotterdam, Netherlands; ^2^Section of Geriatric Medicine, Department of Internal Medicine, Erasmus MC, Rotterdam, Netherlands; ^3^Center for Economic Research, Tilburg University, Tilburg, Netherlands; ^4^Department of Technology and Operations Management, Rotterdam School of Management, Erasmus University, Rotterdam, Netherlands; ^5^Royal Dutch Visio, Huizen, Netherlands

**Keywords:** visuomotor integration, neurodegeneration, eye-hand coordination, biomarker, preclinical AD

## Abstract

**Background:**

Research into Alzheimer’s disease has shifted toward the identification of minimally invasive and less time-consuming modalities to define preclinical stages of Alzheimer’s disease.

**Method:**

Here, we propose visuomotor network dysfunctions as a potential biomarker in AD and its prodromal stage, mild cognitive impairment with underlying the Alzheimer’s disease pathology. The functionality of this network was tested in terms of timing, accuracy, and speed with goal-directed eye-hand tasks. The predictive power was determined by comparing the classification performance of a zero-rule algorithm (baseline), a decision tree, a support vector machine, and a neural network using functional parameters to classify controls without cognitive disorders, mild cognitive impaired patients, and Alzheimer’s disease patients.

**Results:**

Fair to good classification was achieved between controls and patients, controls and mild cognitive impaired patients, and between controls and Alzheimer’s disease patients with the support vector machine (77–82% accuracy, 57–93% sensitivity, 63–90% specificity, 0.74–0.78 area under the curve). Classification between mild cognitive impaired patients and Alzheimer’s disease patients was poor, as no algorithm outperformed the baseline (63% accuracy, 0% sensitivity, 100% specificity, 0.50 area under the curve).

**Comparison with Existing Method(s):**

The classification performance found in the present study is comparable to that of the existing CSF and MRI biomarkers.

**Conclusion:**

The data suggest that visuomotor network dysfunctions have potential in biomarker research and the proposed eye-hand tasks could add to existing tests to form a clear definition of the preclinical phenotype of AD.

## Introduction

Alzheimer’s disease (AD) is a devastating neurodegenerative disease. It is characterized by progressive cognitive decline. It is estimated that it affects 44 million people worldwide (Patterson, 2018). Despite the many attempts to find a cure for AD, no disease-modifying treatment has been developed (Au et al., 2015). Some suggest that experimental treatments were administered too late in the course of the disease (Sperling et al., 2011). Instead, optimal treatment would be achieved in the preclinical stage of AD (Sperling et al., 2011), when pathophysiological changes are already occurring, but no clinical symptoms—such as memory loss—have manifested (Jack et al., 2011). In clinical practice, it is impossible to accurately diagnose patients in the preclinical stage: in fact, a definitive diagnosis can only be established post-mortem (Au et al., 2015). Biomarkers, such as cerebrospinal fluid (CSF) analysis and neuroimaging techniques, are one of the most promising methods for detecting preclinical AD (Barber, 2010). Yet, to date, no biomarker has been identified at the start of the pathophysiological processes of AD, that also has a strong link with the later emergence of AD’s clinical symptoms (Sperling et al., 2011). In addition, given the suboptimal sensitivity and specificity of CSF and structural neuroimaging markers, their use is mostly restricted to research settings (Almeida, 2002; De Leon et al., 2004; Schmand et al., 2010; Ritchie et al., 2015; Jack et al.). Another problem is the heterogeneous nature of AD: a certain level of a biomarker may be detected in some people, even though they will never develop AD, while the same biomarker level might not be detected in people who did develop AD (Au et al., 2015). For these reasons, AD is still primarily diagnosed on clinical grounds (Salimi et al., 2018) and consequently, most patients are not diagnosed until they have progressed to late stages of AD (Barber, 2010).

Recent studies have indicated that multiple functional networks throughout the entire brain are already affected by neurodegeneration in early stage AD (Seeley et al., 2009; Barber, 2010). For instance, there is not only damage to the hippocampus, a structure that is classically associated with the memory impairments in AD, but functional imaging studies showed that other areas, for example in the frontal and parietal lobe, also atrophied (Burnod et al., 1999; Jacobs et al., 2012). This atrophy damages several important networks in the brain: the visuomotor network is one such important function brain network. This network receives visual and proprioceptive sensory to compute motor control signals. The planning and executing of the tasks at hand rely on feedback and feedforward mechanisms through visual, motor, parietal, and frontal cortices (Ledberg et al., 2007) in a dynamic manner (Brovelli et al., 2017). The functionality of the visuomotor network can be quantified in terms of timing, accuracy, and speed of eye and hand movements when performing goal-directed tasks. Consequently, impairments in visuomotor functioning due to underlying neurodegenerative damage could be a marker of early or even preclinical AD and a valuable addition to existing markers (de Boer et al., 2016; Salimi et al., 2018). Several studies support this hypothesis: for instance, it was found that the initiation of saccades was slowed (Molitor et al., 2015; de Boer et al., 2016) and that the initiation and execution of motor sequences was impaired (Salek et al., 2011; de Boer et al., 2016) in AD and MCI patients compared to healthy elderly. Furthermore, visuomotor functioning has the benefit of being easy and quick to measure, compared to existing markers, which are often more time-consuming (i.e., structural neuroimaging) or invasive (i.e., CSF). However, although aforementioned studies show a relation, much is still unclear about the added value of visuomotor performance for network degeneration and the sensitivity and specificity of the method. We aimed to study the predictive value of visuomotor performance by measuring several visuomotor parameters, such as the time needed to move the eyes or the hand to a target. Because only one parameter is not sufficient for a classification (Crawford, 2015), a combination of parameters with high predictive power were analyzed using machine learning. This is a promising method, as it can reduce a large dataset to a small subset of meaningful features using feature selection methods (Sarica et al., 2018). Thus, machine learning provides insight in the clinical relevance (i.e., what visuomotor parameters add to existing markers) of visuomotor performance on the basis of multiple parameters. A first step toward establishing the predictive value was taken by Lagun et al. (2011), who showed that classification algorithm trained on separating healthy elderly and AD patients could distinguish between healthy elderly and MCI patients based on their eye movements, with an accuracy of 87%.

The aim of the present study was to examine whether visuomotor functioning, in terms of both eye and hand movements, can be a potential new biomarker. We also examined whether there was additional predictive value in using eye-hand coordination parameters over just using eye movement parameters (as in Lagun et al., 2011). To this end, we evaluated the performance of several classification algorithms when classifying controls, MCI, AD and consequently determining the predictive power of visuomotor functioning with only eye movements, or both eye and hand movements.

## Materials and Methods

### Participants

Participants were included from June 2010 to January 2018. This study was approved by the Medical Ethical Committee of the Erasmus MC Rotterdam (MEC-2008-365 and MEC 2012-524). Data collected until 2015 were previously published by Verheij et al. (2012) and de Boer et al. (2016). Patients were independently diagnosed using a standardized diagnostic procedure carried out by a panel of specialists from the departments of Geriatrics, Neurology, and Psychiatry from the Erasmus Medical Centre. AD subjects met the standard criteria for probable AD (NINCDS-ADRDA) and were considered to be in early stages of the disease. The clinical diagnosis of MCI and AD was based on patient history, laboratory findings, and imaging results. Family members or caregivers of the patients were recruited as healthy elderly controls. Subjects were excluded if their *mini-mental state examination* (MMSE) (Folstein et al., 1975) score was below 21/30, if they displayed neurological or psychiatric disorders (excluding cognitive decline related to the diagnosis), or if they had motor or ocular pathologies which rendered them unable to touch or see stimuli (unless eyesight could be corrected). The use of acetylcholinesterase inhibitors was permitted. A written informed consent was obtained from all subjects. All collected data were stored in a database.

### Measurement Setup

Eye hand tasks were presented on a 32-inch touchscreen (ELO touch systems). Eye movements were recorded with a head-mounted video infrared eye-tracking system. We used to systems: Chronos, Chronos Vision, Berlin, sampling rate of 200Hz, the EyeSeeCam system, which replaced the Chronos system in 2017 (EyeSeeTec GmbH, Munich, Germany, sampling rate of 220 Hz, resampled to 200 Hz so that the same code could be used to analyze EyeSeeCam and Chronos data). Both systems used pupil detection software. Chronos recorded gaze data of both eyes, whereas EyeSeeCam recorded the gaze data of the left eye. Hand movements were recorded by an infrared motion capture system (Vicon, Vicon Motion, Oxford, sampling rate 200 Hz). Subjects wore a glove with four markers on the dominant hand, which reflected infrared light back to 4 cameras to follow hand movements. All systems were synchronized using a trigger controlled by MATLAB R2017a (MathWorks, Natick, MA). The set-up is illustrated in Supplementary Figure 1.

### Experimental Procedures

All participants were tested at the department of Neuroscience of the Erasmus Medical Centre. Each participant completed a MMSE to assess general cognitive functioning. Subjects were seated at 60 cm distance from the touchscreen using a chin rest. Eye and hand position were calibrated with a 5-point calibration scheme. Verbal instructions were given and subjects were asked to act as quickly and accurately as possible. Subjects performed eight different tasks, three eye tasks, see Supplementary Figure 2, top panel and 5 eye-hand tasks, see Supplementary Figure 2, bottom panel. The location of the presented stimuli was randomized across the screen. Data from the following tasks were selected from the database when available:

(1) Reflex tasks: assessment of reflexive eye movements (pro-saccade task) and eye/hand movements (pro-tapping task).
(2) Inhibition tasks: assessment of inhibitory eye movements (anti-saccade task) and eye/hand tasks (anti-saccade anti-tapping task and anti-tapping task).
(3) Memory tasks: assessment of short-term memory eye movements (memory-saccade task) and eye/hand movements (memory-tapping task and sequential-tapping task).

Each task started with three practice trials, followed by eight measurement trials. First, the eye tasks were presented, followed by the eye-hand tasks. The tasks that involved hand movement were executed with the index finger of the dominant hand. Between 2010 and 2018, different protocols have been applied. Thus not every participant underwent each task. In Supplementary Table 1, an overview is presented of the number of participants per group who completed a given task. Supplementary Figure 3 represents a typical example of eye and hand traces. Data were pre-processed using custom MATLAB code. The following parameters were calculated for eye movements:

- Eye latency: time between target stimulus presentation and eye movement initiation
- Eye maximum velocity: maximum eye movement velocity during the primary saccade.
- Eye error: difference in degrees between target position and final eye position.
- Saccadic amplitude: amplitude of the primary saccade divided by target amplitude.
- Number of saccades: number of saccades made during the trial.

The following parameters were calculated for hand movements:

- Hand latency: time between target stimulus presentation and release of the touchscreen.
- Hand maximum velocity: maximum hand velocity during the hand movement.
- Hand error: difference in degrees between target position and final hand position.
- Hand execution time: time between releasing (click-up) and touching (click-down) the touchscreen.
- Eye hand interval: time between the initiation of the eye and the click-up.
- Eye touch interval: time between the initiation of the eye movement and the click-down.
- Hand total distance: total distance bridged by the hand movement during the trial.
- Amplitude Click-up Click-down: hand movement amplitude between the first click-up and click-down divided by target amplitude.

Finally, several parameters were calculated from the eye trace to quantify pupil characteristics (Wang and Munoz, 2015):

- Pupil peak: maximum pupil dilation between target onset and the primary saccade.
- Pupil latency: time from target onset until maximum pupil dilation occurs.
- Pupil baseline: mean pupil dilation during the fixation period.

Additionally, anticipation (measure of whether the eye movement toward the first and second stimuli preceded the hand movement toward the same stimulus) was calculated for the sequential-tapping task. Finally, a measure of task performance was computed for each task, based on the number of correct trials (all correctly performed trials) divided by the number of valid trials (all trials that could be analyzed). Trials that had not been recorded correctly (invalid trials) were excluded.

### Data Handling

Data from the memory saccade task were excluded because data was collected from too few participants. Outliers—defined as data points that were more than two standard deviations removed from the group mean—were excluded per trial per subject. When eye latency, hand latency, or pupil latency were outliers, the eye, hand, or pupil parameters were all removed, because outliers in these parameters often indicated faulty measurements. In other cases, only the parameter that was an outlier was removed. On average, 4.26% of all data points (*SD* = 2.65%, range: 0–11.99%) were excluded as outliers. Any missing value was marked as “not a number.” Lastly, data were standardized with *z*-scores within the external cross-validation procedure to prevent data leakage. The final dataset consisted of 96 subject instances where each instance contained 77 functional parameters and one nominal variable, which indicated the subject’s group. The dataset was prepared for classification in WEKA 3.8 (WEKA, University of Waikato, New-Zealand). A Zero rule algorithm was used as baseline (*ZeroR* in WEKA). This algorithm predicts that every subject is part of the largest group in the dataset. The performance of three supervised algorithms was compared to this baseline:

1. *Support vector machine* (SVM) (Cortes and Vapnik, 1995): a linear classifier which separates groups at the point where the distance between the groups is the greatest by drawing a hyperplane. SVMs use the *kernel trick* (Shawe-Taylor and Cristianini, 2004) to perform non-linear classification: data are transformed, using a mapping function (defined by the kernel), to a high-dimensional space where a linear hyperplane can be found. When this hyperplane is transformed back to 2-dimensional space, it will become a non-linear separator. A drawback of the kernel trick is that the most effective type of kernel is not known beforehand, making it necessary to test multiple kernels (*LibSVM* function in WEKA). The SVM was chosen in this study so a direct comparison could be made with Lagun et al.’s study (2011), who looked at the value of eye movement parameters for distinguishing between healthy controls and MCI or AD patients. Furthermore, SVMs are fast to train and are flexible because different kernels can be included in the model. We included a variety of kernels (radial basis function kernel, polynomial kernel, sigmoid kernel, linear kernel) to accommodate potential differences in data structures and to compare the performance of simple and high dimensional kernels. Although better performance might be expected from higher dimensional kernels, simpler kernels save time and processing power: therefore, there is value in comparing the performance of simpler kernels to higher dimensional kernels.
2. *Decision tree* (Breiman et al., 1984): a simple algorithm that splits the dataset into smaller partitions until it can correctly classify most instances. For each split, the parameter that would provide the best separation between groups at that point is chosen. The decision tree was selected because it is a simple, easily interpretable, and computationally inexpensive algorithm that gives immediate insight in the parameters and weights used for classification (*J48* algorithm in WEKA).
3. *Artificial neural network* (ANN) (Schmidhuber, 2015): the algorithm creates a network of hidden nodes, with differing weights, which are activated or deactivated depending on the input they receive. The activation pattern of all nodes is transformed into the predicted class for the subject. The ANN is a very flexible algorithm, that performs well on complex problems (*multilayer perceptron* in WEKA). ANNs are also suitable to handling multiclass problems, which the SVM does not readily lend itself for.

We selected these three models for comparison and to find the model with the best performance and the easiest implementation. For example, the ANN is expected to do well because it is tailored to complex problems, but it is computationally heavy and takes a long time to train. Therefore, if a decision tree or SVM could achieve similar performance, these types of algorithms would be preferred in practice. This is especially true for the decision tree, as this algorithm gives complete insight in which features are used. However, since the decision tree has more trouble with complex problems, we also included the SVM as the middle road.

**FIGURE 1:**
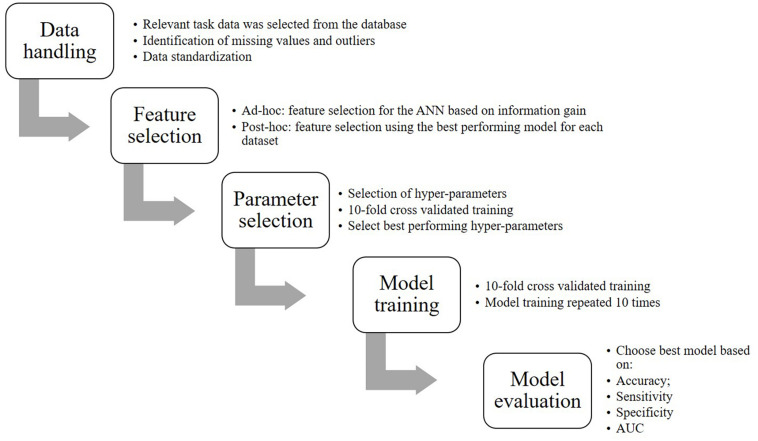
Flowchart of the classification process.

Figure 1 represents a flow chart of the steps that were involved to convert the data into the final model including:

#### Feature Selection

Feature selection was performed for all dataset views to identify the functional variables that would be most meaningful for classification and additionally, to prevent model overfitting by reducing the number of features that the ANN would be trained on. Additionally, we performed significance tests in SPSS 24 (IBM SPSS, Chicago, United States) to verify whether the selected variables could meaningfully separate the groups. Normality was tested for each selected variable using the Shapiro-Wilk test. For normally distributed data, two-tailed *t*-tests were performed and Cohen’s *d* was calculated as a measure of effect size. For non-normally distributed data, a non-parametric Mann-Whitney *U*-test was performed and *r*-squared was calculated as a measure of effect size (Rosenthal et al., 1994). One variable (Anticipation) recorded the number of trials that the subject anticipated a stimulus in the sequential-tapping task and therefore, a chi-square test was performed. *Phi* was taken as a measure of effect size. The significance level for all tests was α = 0.05. Exact *p*-values were reported, unless *p* < 0.001.

To gain insight in the parameters used by models other than the ANN, we performed *post hoc* feature selection in WEKA using the most successful classifier with the same hyperparameter settings on the HC-controls, HC-MCI, HC-AD, and MCI-AD comparisons. This method returned the merit of a parameter and ranked them from most to least merit. Parameters were selected if their merit was > 0.01.

#### Hyper-Parameter Selection

Cross-validated parameter selection was performed to optimize each model’s hyper-parameters. Hyper-parameters influence the learning process of a model. For parameter selection, the hyper-parameters were varied over a pre-established range. All trained instances were compared and the hyper-parameters of the instance with the highest accuracy were chosen. The hyper-parameter that was specified first was optimized first and with that optimal value, the second hyper-parameter was optimized. Optimization was internally cross-validated 10 times. Specifically, for the ANN we optimized learning rate and momentum (ranges: 0.0001–1); for the decision tree we optimized the confidence factor (range: 0.1–0.9) and the minimum number of objects (range: 1–10); and for the SVM we optimized the cost and the gamma (ranges: 0.0001–1).

### Model Training

Eight optimized models were trained for each dataset view: one decision tree, three ANNs with one hidden layer with 2, 4, or 6 hidden neurons, and four SVMs with different kernels (radial basis function kernel, polynomial kernel, sigmoid kernel, linear kernel). Model training was performed in WEKA with 10-fold cross-validation. The model training was repeated 10 times in order to achieve more robust results.

#### Model Evaluation

Classification performance was assessed based on accuracy (correctly classified data points/total data points), sensitivity (data points correctly classified as positives/total data points classified as positives), specificity (data points correctly classified as negatives/total data points classified as negatives), and area under the curve (AUC, measure of classification accuracy, independent of class distribution). These outcome measures were averaged across the 100 repetitions and reported as the final performance estimates. Per dataset view, the model that scored the highest on most performance metrics, as determined with a one-way analysis of variance (ANOVA), was selected as the “best performing model.” When there was no significant difference in performance between models (α = 0.05), models with a higher sensitivity were preferred, because it is more important to correctly classify patients. The performance of the “best performing model” was assessed using the following rules of thumb. These rules did not apply to the baseline algorithm, which was considered to be a poor performer in all cases because of its strategy. Thus, algorithms that performed similarly to the baseline were also considered poor performers.

### Learning Curves

We generated learning curves in WEKA to assess whether sample size was sufficient. Learning curves are used to indicate how much data a model needs to be trained on to achieve a good performance (Figueroa et al., 2012). The curves were generated by plotting model accuracy against sample size. Sample size was manipulated by training the algorithm on a fraction of the total dataset (starting at 10%), which was increased by 10% until the complete dataset was used. Typically, a learning curve that indicates a sufficient sample size first shows a rapid increase in performance, followed by a turning point where the performance increase slows. The last part of the curve is flat and indicates that performance will not improve further, even if sample size is increased (Figueroa et al., 2012).

## Results

**FIGURE 2:**
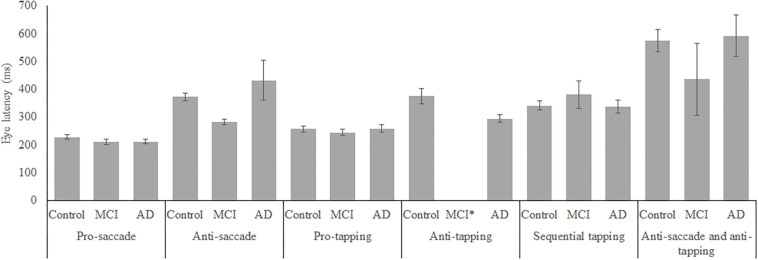
Mean performance on eye latency for all groups, on all tasks. Error bars represent standard error. MCI*: MCI group was excluded because of insufficient data (*N* = 3).

**FIGURE 3:**
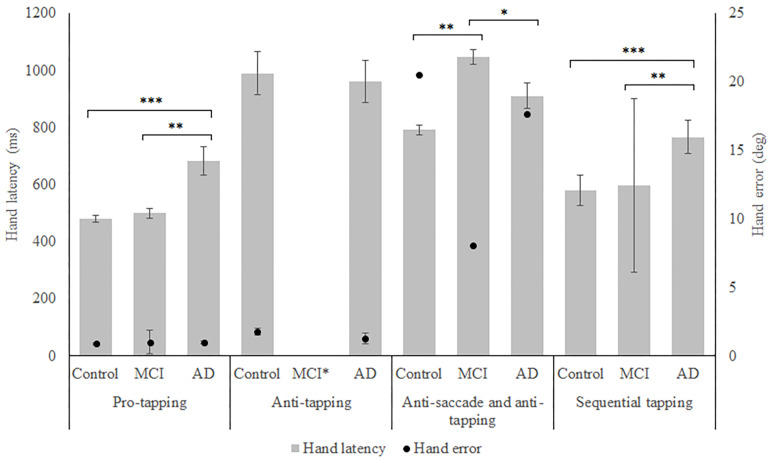
Mean performance on hand latency and hand error for all groups, on all tasks. Error bars represent standard error. Significant differences for the anti-saccade anti-tapping task indicate differences for hand error; other indicated differences are for hand latency. Significant differences were indicated with ^∗^*p* < 0.05, ^∗∗^*p* < 0.01, ^∗∗∗^*p* < 0.001. MCI^∗^: MCI group was excluded because of insufficient data (*N* = 3).

**FIGURE 4:**
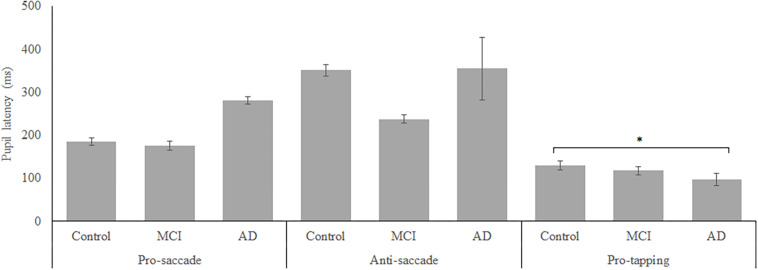
Mean performance on pupil latency for all groups. Error bars represent standard error. Significant differences were indicated with ^∗^*p* < 0.05, ^∗∗^*p* < 0.01, ^∗∗∗^*p* < 0.001.

**FIGURE 5:**
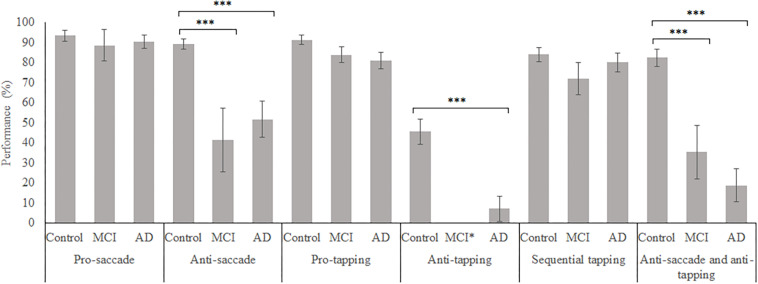
Mean weighted performance over all trials for all groups, on all tasks. Error bars represent standard error. Significant differences were indicated with ^∗^*p* < 0.05, ^∗∗^*p* < 0.01, ^∗∗∗^*p* < 0.001. MCI*: MCI group was excluded because of insufficient data (*N* = 3).

In total, 96 subjects were selected from the database. Table 1 provides an overview of the demographics of these subjects. 21 MCI patients were diagnosed with MCI due to Alzheimer’s disease and 1 was diagnosed with MCI or early stage dementia. The mean age of all subjects was 74.9 years (SD = 6.15) and the mean MMSE score was 25 (*SD* = 4.01). A one-way ANOVA showed no differences between groups in age [*F*_(2, 91)_ = 1.097, *p* = 0.338] and a difference in MMSE scores between groups [*F*_(2, 83)_ = 23.37, *p* < 0.001]. *Post hoc* tests, corrected for multiple comparisons with a Bonferroni correction, showed that the mean MMSE scores of the controls were higher than of the MCI patients (*p* < 0.001) or the AD patients (*p* < 0.001). Figures 2–5 give an overview of the raw performance data of each group (Figure 2: eye latency; Figure 3: hand latency, and hand error; Figure 4; pupil latency; Figure 5: performance).

**TABLE 1:** Subject demographics.

Parameter	Controls	MCI	AD
N	37	22	37
Male	22 (59.45%)	12 (54.55%)	20 (54.05%)
Female	15 (39.55%)	10 (45.45%)	17 (55.95%)
Age	75.9 (6.3)	75.5 (7.1)	73.7 (4.77)
Age range	65–84	65–90	63–88
MMSE	29 (1.86)	24 (2.79)	23 (4.28)

### Feature Selection

Table 2 presents the information gain (if selected for the ANN) or merit (if selected by the SVM) and the results of the significance tests for each selected variable. The variables are divided per task to provide better insight in the importance of each task. For illustration purposes, we chose to present the results of the significance tests only for the HC and patients (both MCI and AD) dataset. All selected variables differed significantly between the control group and the aggregated patient group and most of the variables showed medium to very large effect sizes (Cohen, 1988). To give an indication of the similarities between the selected variables between datasets the feature selection results for the controls—MCI, controls—AD, and MCI—AD datasets, using all parameters are presented in Table 2. Table 3 reports the descriptive statistics of the functional variables that were selected based on their information gain (gain > 0), except for the variables that were only selected for the MCI—AD dataset, as these variables did not provide sufficient predictive power to classify MCI and AD patients.

**TABLE 2:** The feature selection results, ranked by IG and merit, for the HC—MCI, HC—AD, and MCI—AD datasets.

HC—MCI		HC—AD		MCI—AD	
		
Variables	IG	Variables	IG	Variables	IG
Hand movement time (stimulus 1) (ST)***	0.187	Hand latency (PT)***	0.190	Hand latency (PT)***	0.165
Hand movement time (stimulus 2) (ST)***	0.157	Hand latency (stimulus 2) (ST)***	0.186	Pupil latency (AS)	0.028
Eye touch interval (stimulus 1) (ST)***	0.110	Hand movement time (stimulus 1) (ST)***	0.161	Hand total distance (ASAT)	0.005
Eye touch interval (stimulus 2) (ST)	0.077	Hand movement time (PT)	0.132	Hand error (AT)	0.004
Hand error (ASAT)**	0.048	Eye-hand interval (PT)***	0.126	Hand movement time (AT)	0.004
Eye latency (ASAT)	0.034	Hand latency (stimulus 1) (ST)***	0.118	Hand latency (AT)	0.004
		Eye latency (AS)	0.113	Hand maximum velocity (ASAT)*	0.003
		Hand movement time (stimulus 2) (ST)***	0.108	Hand maximum velocity (AT)	0.002
		Eye touch interval (stimulus 1) (ST)***	0.097		
		Pupil latency (AS)	0.077		
		Saccadic error (PS)***	0.067		
		Anticipation (stimulus 2) (ST)	0.016		

**HC—MCI (SVM)**		**HC—AD (SVM)**			
	
**Variables**	**Merit**	**Variables**	**Merit**		

Number of saccades (PT)	0.034	Hand latency (PT)***	0.030		
Hand latency (AT)	0.027	Hand latency (AT)	0.022		
Hand total distance (ASAT)*	0.020	Number of saccades (PT)	0.022		
Hand maximum velocity (ASAT)*	0.014	Pupil latency (AS)	0.016		
Hand movement time (AT)	0.014	Eye-hand interval (PT)***	0.014		
Hand error (PT)	0.014	Eye latency (AS)	0.014		
Eye-hand interval (stimulus 1) (ST)***	0.014	Pupil latency (PT)*	0.011		
Number of saccades (PS)	0.014	Amplitude Click up-Click down (PT)*	0.011		
Fixation error (PS)	0.010				
Eye latency (PT)	0.010				
Saccadic error (PS)***	0.010				
Amplitude Click up- Click down (PT)*	0.010				

See Figures 1, 2 for the task name abbreviations. * indicates the variable was significantly different between the groups in that dataset (*p ≤ 0.05; **p ≤ 0.01, ***p ≤ 0.001).

**TABLE 3:** Descriptive statistics of the most important parameters per task.

EHC task	Variable	HC (mean ± SD)	MCI (mean ± SD)	AD (mean ± SD)
Pro-saccade	Fixation error	1.8 ± 0.7*deg*	2.7 ± 1.3*deg*	2.7 ± 1.7*deg*
	Saccadic error	3.6 ± 1.3*deg*	4.5 ± 1.6*deg*	5.2 ± 1.8*deg*
	Number of saccades	2.7 ± 0.9	3.1 ± 1.0	3.1 ± 1.4
Anti-saccade	Saccadic error	10.1 ± 3.7*deg*	17.0 ± 4.3*deg*	14.0 ± 6.2*deg*
Pro-tapping	Eye-hand interval	240 ± 66*ms*	251 ± 75*ms*	390 ± 233*ms*
	Hand movement time	518 ± 147*ms*	637 ± 217*ms*	603 ± 163*ms*
	Amplitude click up—click down	25.6 ± 1.44^m^	25.1 ± 2.2^m^	24.5 ± 2.1^m^
	Number of saccades	2.2 ± 0.9	2.0 ± 0.7	2.2 ± 0.8
Anti-tapping	Hand total distance	138 ± 51*mm*	*	166 ± 50*mm*
	Hand movement time	716 ± 285*ms*	*	877 ± 371*ms*
	Hand maximum velocity	740 ± 953^v^	*	538 ± 201^v^
Sequential tapping	Hand total distance (stimulus 1)	15.9 ± 7.2*mm*	24.4 ± 21.8*mm*	16.1 ± 4.2*mm*
	Eye-hand interval (stimulus 1)	248 ± 74*ms*	305 ± 108*ms*	342 ± 140*ms*
	Eye touch interval (stimulus 1)	730 ± 115*ms*	967 ± 178*ms*	900 ± 284*ms*
	Hand movement time (stimulus 1)	477 ± 79*ms*	729 ± 178*ms*	644 ± 193*ms*
	Hand movement time (stimulus 2)	506 ± 100*ms*	663 ± 165*ms*	596 ± 152*ms*
	Anticipation (stimulus 2)	Range: 1–2 trial(s)	1 trial(s)	1 trial(s)
Anti-saccade anti-tapping	Hand maximum velocity	496 ± 106^v^	*	459 ± 183^v^
	Hand total distance	104 ± 46	82 ± 82	153 ± 31

*Only 3 MCI patients had valid data and were there excluded from all analyses for these measures.

### Predictive Power: Controls vs. Patients

Table 4 displays the performance of all algorithms on the controls and patients dataset. The baseline algorithm achieved a sensitivity of 100% and a specificity of 0%, because it predicted all subjects were patients. Whether sensitivity or specificity was 100% depended on which group was the largest, as the baseline algorithm predicted that all instances were part of the largest group. To assess which algorithm performed the best, a one-way ANOVA was performed for each performance metric. For all metrics, the mean performance of the tested algorithms differed significantly [accuracy: *F*_(8, 891)_ = 47.41, *p* < 0.001, partial η^2^ = 0.30; sensitivity: *F*_(8, 891)_ = 85.96, *p* < 0.001, partial η^2^ = 0.44; specificity: *F*_(8, 891)_ = 139.08, *p* < 0.001, partial η^2^ = 0.56; AUC: *F*_(8, 891)_ = 83.40, *p* < 0.001, partial η^2^ = 0.43]. Additional *post hoc* tests revealed that the SVM (linear kernel) had the best accuracy, the SVM (sigmoid) the best sensitivity, the SVM (linear) the best specificity and the decision tree had the best AUC. Overall, the SVM (linear) performed best or amongst the best on all metrics and it achieved a good classification. The confusion matrix of this algorithm is shown in Table 5.

**TABLE 4:** Performance of all algorithms for the classification of controls and patients, ordered by descending accuracy per algorithm class.

Algorithm	Accuracy	Sensitivity	Specificity	AUC
Baseline (Zero rule)	62%	100%	0%	0.50
ANN (2 neurons)	72 ± 14%	77 ± 17%	62 ± 25%	0.76 ± 0.16
ANN (4 neurons)	70 ± 14%	74 ± 16%	63 ± 25%	0.76 ± 0.16
ANN (6 neurons)	69 ± 14%	74 ± 16%	62 ± 24%	0.76 ± 0.16
Decision tree	76 ± 12%	88 ± 13%	57 ± 24%	0.82 ± 0.15
**SVM (linear kernel)**	**82 ± 11%**	**93 ± 10%**	**63 ± 27%**	**0.78 ± 0.16**
**SVM (polynomial kernel)**	**81 ± 12%**	**94 ± 11%**	**60 ± 28%**	**0.77 ± 0.14**
SVM (RBF kernel)	80 ± 11%	97 ± 8%	53 ± 26%	0.75 ± 0.13
SVM (sigmoid kernel)	62 ± 4%	100 ± 0%	0 ± 0%	0.50 ± 0

*The abbreviation RBF stands for radial basis function kernel.*

*The two models in bold are the best performing models.*

**TABLE 5:** Confusion matrix for the performance of the SVM (linear) for the controls-patients comparison.

	Controls (predicted)	Patient (predicted)
Controls (actual)	63%	37%
Patient (actual)	7%	93%

**The result of the hyperparameter tuning for the SVM (linear) was a cost of 0.316. Cost represents the penalty given for misclassification: a higher cost allows less misclassification when drawing the decision boundary.**

### Predictive Power: Controls vs. MCI/AD and MCI vs. AD

#### Control vs. MCI

The means differed significantly for all metrics [accuracy: *F*_(8, 891)_ = 19.49, *p* < 0.001, partial η^2^ = 0.15; sensitivity: *F*_(8, 891)_ = 77.10, *p* < 0.001, partial η^2^ = 0.41; specificity: *F*_(8, 891)_ = 70.05, *p* < 0.001, partial η^2^ = 0.39; AUC: *F*_(8, 891)_ = 31.91, *p* < 0.001, partial η^2^ = 0.22]. *Post hoc* analyses, corrected for multiple comparisons using the Bonferroni test, indicated that the SVM (linear) was the best performing algorithm on all metrics (see also Table 6).

**TABLE 6:** Performance of the best performing algorithm, with hyperparameter selection results, for the classification of controls and MCI Patients, controls and AD Patients, and MCI Patients and AD patients with all data and separately for eye tasks and eye-hand tasks.

Dataset	Algorithm	Accuracy	Sensitivity	Specificity	AUC
Controls—MCI	Baseline	63%	0%	100%	0.50
	SVM (linear) (*C* = *0.579*)	77 ± 16%	57 ± 34%	90 ± 16%	0.74 ± 0.18
Controls—AD	Baseline	46%	30%	70%	0.50
	SVM (linear) (*C* = *0.369*)	78 ± 16%	71 ± 22%	84 ± 20%	0.78 ± 0.16
MCI—AD	Baseline	63%	0%	100%	0.50
	ANN (6 neurons) (*Default*)	61 ± 17%	39 ± 39%	75 ± 25%	0.66 ± 0.25
HC—MCI	Baseline	63%	0%	100%	0.50
Eye tasks	SVM (linear)	64 ± 8%	3 ± 11%	100 ± 0%	0.51 ± 0.50
Eye-hand tasks	SVM (linear)	77 ± 18%	60 ± 31%	88 ± 17%	0.74 ± 0.19
HC—AD	Baseline	46%	30%	70%	0.50
Eye tasks	SVM (linear)	70 ± 13%	43 ± 25%	96 ± 10%	0.70 ± 0.13
Eye-hand tasks	SVM (linear)	81 ± 14%	76 ± 21%	86 ± 17%	0.81 ± 0.14

#### Control vs. AD

The means differed significantly for all metrics [accuracy: *F*_(8, 891)_ = 45.07, *p* < 0.001, partial η^2^ = 0.29; sensitivity: *F*_(8, 891)_ = 19.15, *p* < 0.001, partial η^2^ = 0.15; specificity: *F*_(8, 891)_ = 4.59, *p* < 0.001, partial η^2^ = 0.04; AUC: *F*_(8, 891)_ = 38.68, *p* < 0.001, partial η^2^ = 0.26]. *Post hoc* analyses, using the Bonferroni test, indicated that the SVM (linear) was the best performing algorithm on accuracy; on the other metrics, all algorithms performed similarly, except the baseline algorithm and the SVM (sigmoid), which performed worse.

#### MCI vs. AD

the means differed significantly for all metrics [accuracy: *F*_(8, 891)_ = 5.55, *p* < 0.001, partial η^2^ = 0.05; sensitivity: *F*_(8, 891)_ = 53.30, *p* < 0.001, partial η^2^ = 0.32; specificity: *F*_(8, 891)_ = 71.40, *p* < 0.001, partial η^2^ = 0.39; AUC: *F*_(8, 891)_ = 24.82, *p* < 0.001, partial η^2^ = 0.18]. *Post hoc* analyses, using the Bonferroni test, indicated that the ANN (6 neurons) performed best on sensitivity, and AUC. On accuracy, the ANN did not outperform the baseline algorithm. On specificity, the ANN was outperformed because algorithms behaving like the baseline algorithm showed 100% specificity. These algorithms were disregarded, leaving the ANN (6 neurons) as the algorithm with the highest sensitivity.

The confusion matrices of these comparisons are shown in Table 7.

**TABLE 7:** Confusion matrices of the SVM (linear) for the controls and MCI patients classification and the controls and AD patients classification, and the ANN (6 neurons) for the MCI patients and AD patients classification.

	Controls (predicted)	MCI (predicted)	AD (predicted)
Controls (actual)	90%	10%	
MCI (actual)	43%	57%	
Controls (actual)	84%		16%
AD (actual)	29%		71%
MCI (actual)		57%	43%
AD (actual)		31%	69%

### Learning Curves

**FIGURE 6:**
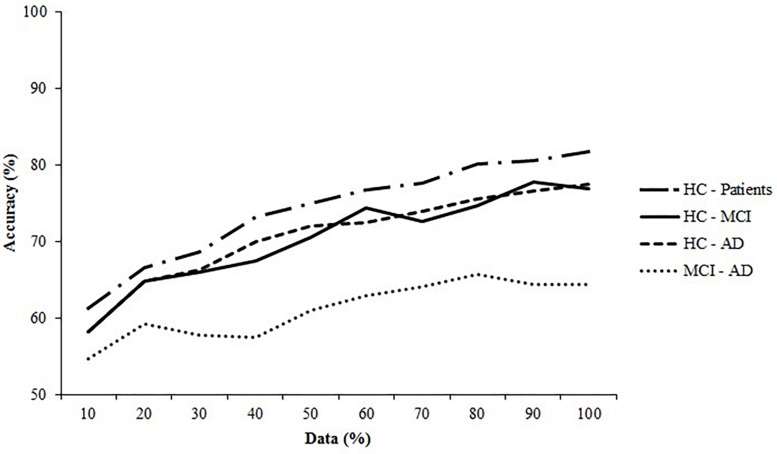
Learning curves for the healthy controls (HC) and the Alzheimer’s Disease (AD) patients and the Mild Cognitive Impairment (MCI) patients. The SVM (linear) for the HC—patients, HC—MCI, and HC—AD are plotted and of the ANN (6 neurons) the MCI—AD dataset is plotted.

Figure 6 shows the learning curves generated for all datasets, using the accuracy of the best performing algorithm plotted against sample size. The learning curves for the controls and patients, controls and MCI patients, and controls and AD patients show the initial large increase in accuracy and the turning point where this increase slows. The final phase, where performance plateaus, was not achieved. The learning curve for the MCI and AD dataset displayed only a marginal increase in performance, indicating that the algorithm learned minimally from the EHC parameters.

### Predictive Power: Eye Parameters vs. Eye-Hand Parameters

The best performing algorithm for each comparison was trained on one dataset containing all data from the eye tasks and one dataset containing all data from eye-hand tasks. Classification performance was examined for the HC and MCI and the HC and AD datasets. The HC and the patient’s dataset was disregarded because we aimed to directly examine classification separately for the patient groups. The MCI and AD dataset was disregarded because classification failed using all EHC parameters, which indicated that classification would also fail on the splitted datasets. A one-way ANOVA was performed on all metrics to compare classification performance on the split datasets (significance level α = 0.05). The difference in performance was taken as the additional predictive value of eye-hand tasks. Table 4 reports the performance of the SVM (linear) on the split datasets, with the performance of the baseline for reference.

#### Control vs. MCI

Classification based on eye-hand tasks outperformed classification based on eye tasks on accuracy, sensitivity, and AUC [accuracy: *F*_(1, 198)_ = 48.69, *p* < 0.001, partial η^2^ = 0.20; sensitivity: *F*_(1, 198)_ = 300.03, *p* < 0.001, partial η^2^ = 0.60; AUC: *F*_(1, 198)_ = 129.28, *p* < 0.001, partial η^2^ = 0.40]. Classification based on eye tasks performed better on specificity [*F*_(1, 198)_ = 51.62, *p* < 0.001, partial η^2^ = 0.21]; however, the confusion matrix of this algorithm (Table 8) suggests that it performed similarly to the baseline and only had a high specificity at the expense of the sensitivity. Therefore, classification based on eye-hand tasks was considered superior on all metrics.

**TABLE 8:** Confusion matrix of the SVM (linear) for the HC and MCI patients and the HC and AD patients classification using only eye tasks.

	HC (predicted)	MCI (predicted)	AD (predicted
HC (actual)	100%	0%	
MCI (actual)	98%	2%	
HC (actual)	96%		4%
AD (actual)	57%		43%

#### Control vs. AD

Classification based on eye-hand tasks outperformed classification based on eye tasks on accuracy, sensitivity, and AUC [accuracy: *F*_(1, 198)_ = 36.32, *p* < 0.001, partial η^2^ = 0.16; sensitivity: *F*_(1, 198)_ = 104.42, *p* < 0.001, partial η^2^ = 0.35; AUC: *F*_(1, 198)_ = 36.41, *p* < 0.001, partial η^2^ = 0.16]. Classification based on eye tasks performed better on specificity [*F*_(1, 198)_ = 26.70, *p* < 0.001, partial η^2^ = 0.12]; however, the confusion matrix of this algorithm (Table 8) suggests that the high specificity and lower sensitivity followed the pattern of the baseline algorithm. Therefore, classification based on eye-hand tasks was considered superior on all metrics.

## Discussion

In the present study, the functional consequences of neurodegeneration on the visuomotor network have primarily been assessed in both patients with AD and in patients with mild cognitive impairments using eye-hand coordination (EHC) tasks. The aim of this study was to establish the predictive value of EHC for network dysfunction and the sensitivity and the specificity of this method. This predictive power depended on the predictive power of the individual parameters that constitute visuomotor performance, such as the timing, accuracy and speed of movements. An accuracy of 87% was found using a classification algorithm that distinguished between healthy elderly and MCI patients, based on only eye movement parameters (Lagun et al., 2011). In the present study, a combination of eye and hand movements parameters were obtained, because AD patients seem to have difficulties with handling increased load (Toepper, 2017). As expected, the best classification performance was achieved for HC and AD patients, followed by HC and all patients, and finally HC and MCI patients. The eye-hand task parameters, in comparison with only eye movement parameters, were shown to contribute most to classification performance for all comparisons (Tables 2, 4). Based on the eye tasks, we found a reduction in predictive power of ± 12%. This indeed suggests that an increase in cognitive load contributes to the best classification of controls and either patient group.

The predictive power of visuomotor performance was determined by several parameters that were selected during the feature selection procedure using the best performing model for each comparison, to gain insight in the parameters that were most useful for classification (Tables 2, 4). The parameters with the most merit for classification primarily came from the reflexive tapping task and from the inhibition tapping task, contributing to the hypothesis that adding hand movement to the tasks increases their predictive value. In particular, parameters related to latency (time between target presentation and movement initiation), parameters that characterize hand movement (e.g., hand total distance, hand maximum velocity, hand movement time, hand error), and the number of saccades were the most useful information for classification. Several of these parameters have also been described in previous EHC studies (Molitor et al., 2015). For instance, the finding that reflexive saccades made by AD patients are often hypometric is comparable to the parameter *saccadic error* (the distance between the end position of a saccade and the actual target position) which was selected in this study. Another example is the increased latency of eye movements when executing the anti-saccade task. Furthermore, it was found that the initiation and execution of motor sequences were impaired in AD patients and in MCI patients when cognitive load was increased (Salek et al., 2011). We detected a similar impairment in our patient group, represented by the parameter from the pro-tapping task. The remaining selected parameters (Table 2 and Supplementary Table 2) in the current study could not be related to previous literature, as studies into visuomotor performance in MCI and AD patients are scarce. However, the similarities discussed here do support the results of the feature selection procedure and provide additional evidence that these specific tasks and parameters contribute to the predictive power.

**FIGURE 7:**
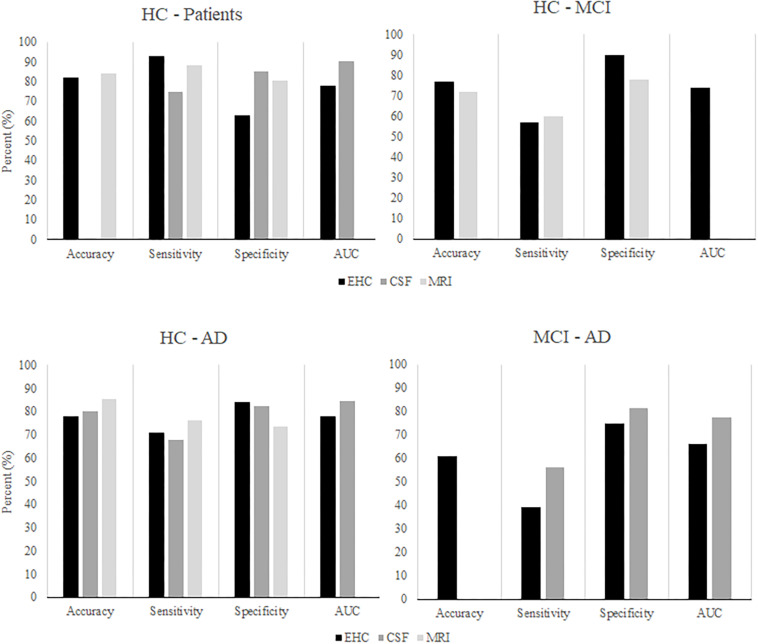
After Henriques et al. (2018). The classification performance of algorithms using CSF or MRI markers to classify HC, MCI patients, and AD patients, compared to classification performance using EHC parameters.

Considering our findings, visuomotor performance appears as a promising approach to distinguish MCI or early stage AD patients from controls. Yet, it must also be compared to existing biomarkers to determine its clinical relevance. The findings of the current study were compared to a recent overview of studies that examined the performance of CSF and neuroimaging markers for the classification of controls, MCI patients, and AD patients (Figure 7; Henriques et al., 2018). Overall, the classification performance found in the present study seems comparable to that of the existing CSF and MRI biomarkers. Unfortunately, our findings are also similar in that neither visuomotor performance nor CSF markers are particularly successful in distinguishing between MCI patients and AD patients. Although the CSF marker classification achieved a fair AUC, sensitivity remained insufficient. In summary, visuomotor performance can compare to the existing biomarkers, although the classification of MCI and AD patients remains challenging. Assessment of visuomotor performance offers some practical advantages: it is simple and quick to perform, non-invasive, and relatively cheap when compared to, for example, MRI scans.

This study has several limitations. First, the predictive power of visuomotor performance, and thus its clinical relevance, depend on the tasks that are included in the eye-hand coordination protocol. Our current protocol focused on reflexive behaviors and inhibition and to a lesser extent on memory. The performance scores on the memory tasks were for a majority of the patients very low. Most of the patients failed to complete the memory tasks, indicating that subjects were too fatigued to properly perform these tasks at the end of the protocol, or that these tasks were not appropriate for this population. Still, memory tasks cannot be disregarded in general (Lagun et al., 2011). These tasks involve the recognition of a previously seen image, a function that relies on a temporal-parietal memory network (Hirose et al., 2013). Interestingly, this network can also be related to the parietal lobe damage that patients sustain in early stages of AD (Jacobs et al., 2012). Because early stage neurodegeneration damages the parietal lobe, this network would likely also be disrupted (Hirose et al., 2013). Therefore, a recognition task could be an adequate addition to our protocol. Previous literature also suggests that a smooth pursuit task might contribute to classification, as studies reported that AD patients showed increased latencies when they initiated smooth pursuit as compared to controls. Additionally, while following the target, patients showed a lower gain and velocity of eye movements, and because patients tended to lag behind the stimulus, they made more catch-up saccades (Molitor et al., 2015).

Second, we performed no power calculations before the study was conducted, because it was an exploratory study. Instead, we assessed sample size *post hoc* by generating learning curves (Figure 2). The learning curves for the SVM on the controls and patients, controls and MCI patients, and controls and AD patients datasets indicate that classification would likely have benefitted from an increased sample size, as the curves do not plateau. An increase in sample size could also benefit model stability: the standard deviations of the performance metrics are large and could indicate noisy data, which could be remedied by increasing sample size. However, the learning curves do reach a point where performance only continues to increase minimally. This indicates that a larger sample size would only have a small effect, as the model has learned most of the important patterns in the data. Therefore, we advise that future studies increase the sample size, as that would likely improve classification performance.

Furthermore, due to the relatively small sample size in this study, there was a discrepancy between the distribution of the control and the patient groups and the distribution of these groups in clinical practice. In our study, controls and AD patients made up an equal part of our sample, whereas in the clinic, most people would be patients: consequently, the group of controls would be relatively small. Even if groups of patients with other types of dementia would be included, 60–80% of the total patient group would still be an AD patient (Williams and First, 2013). This difference is important to keep in mind, because group distribution affects how a model classifies a subject. If almost all instances presented to a model are AD patients and not controls, the algorithm might learn that it can simply classify every subject as being part of the largest group, as it will still achieve a high accuracy. Such a strategy is useless for diagnosis and therefore, it would also be important for future studies to assess the classification models from this study in a more realistically distributed sample. Lastly, data on how subjects performed on conventional diagnostic methods and clinical information such as age of disease onset were unfortunately not available for the complete group of patients. This limits insights in for example how uniform the patient groups were and in which stage of the disease individual patients were. In future studies we recommend that this information is also collected.

As stated previously, early diagnosis is primarily hindered by the heterogeneity of AD, as some patients may show severe symptoms, but no abnormalities in biomarker measurements, or vice versa. As such, no single marker has been identified that can diagnose all cases of AD. It has recently been suggested that AD is not one disease, but a collection of subtypes (Au et al., 2015) that differ in their neuropathological and structural characteristics (Ferreira et al., 2017). The idea of subtypes with different underlying pathologies also gives rise to the hypothesis that an assay of biomarkers could be more useful for diagnosis than relying on a single biomarker, provided that each marker adds new information. For example, it was shown that MRI can complement the standard diagnostic procedure by distinguishing four types of AD using MRI scans, which could not be distinguished using routine cognitive evaluations or CSF analysis (Ferreira et al., 2017). Recent studies have also shown that combining existing biomarkers increases the predictive power beyond what the individual markers could achieve (Zhang et al., 2011; Henriques et al., 2018). Visuomotor performance could provide complementary information (i.e., about functional deficits in patients) in much the same way and when combined with the existing markers, it could further boost the predictive power for early stage AD. As such, testing of visuomotor network dysfunctions could potentially be added to the current diagnostic procedure.

Future research should aim to improve the classification performance of visuomotor network dysfunctions, by adding different types of tasks to develop an optimal protocol. Subsequently, it would be valuable to study whether visuomotor performance correlates with functional network degeneration as measured by neuroimaging techniques, as it is hypothesized to reflect network degeneration. If visuomotor performance can be related to neurodegeneration in this way, next steps should involve replicating the current results in different medical centers and in larger datasets, preferably with a realistic group distribution. Lastly, future research could investigate whether visuomotor functioning could also be a marker for other types of dementia. Neurodegeneration in other dementias also disrupts multiple pathways which are integral to good visuomotor functioning. For example, fronto-temporal dementias could be a valuable field of study, as the frontal lobe is also an important structure for visuomotor function (e.g., planning saccades).

## Conclusion

Visuomotor network dysfunctions have potential in biomarker research and the proposed eye-hand tasks could add to a clear definition of the preclinical phenotype of AD.

## Data Availability Statement

The raw data supporting the conclusions of this article will be made available by the authors, without undue reservation.

## Ethics Statement

The studies involving human participants were reviewed and approved by the Medical Ethical Committee of the Erasmus MC Rotterdam. The patients/participants provided their written informed consent to participate in this study. Written informed consent was obtained from the individual(s) for the publication of any potentially identifiable images or data included in this article.

## Author Contributions

JuS selected, analyzed, and interpreted the data and was a major contributor to writing the manuscript. FM-R designed the study, supported patient recruitment, and interpreted the data. HD advised on the application of the machine learning methodology. JoS designed the eye-hand coordination tasks and interpreted the data. JP designed the study and interpreted the data and was a major contributor in writing the manuscript. All authors read and approved the final manuscript.

## Conflict of Interest

The authors declare that the research was conducted in the absence of any commercial or financial relationships that could be construed as a potential conflict of interest.

## Abbreviations

AD: Alzheimer’s disease
ANN: Artificial neural network
ANOVA: Analysis of variance
AUC: Area under the curve
CSF: Cerebrospinal fluid
EHC: Eye-hand coordination
MCI: Mild cognitive impairment
MMSE: Mini mental state examination
MRI: Magnetic resonance imaging
PS: Pro-saccade
SD: Standard deviation
SVM: Support vector machine.
