# Supplementary material for: To Explore the Predictive Power of Visuomotor Network Dysfunctions in Mild Cognitive Impairment and Alzheimer’s Disease
Source: Front Neurosci. 2021 Jun 28;15:654003. doi: 10.3389/fnins.2021.654003 (PMC8273577; doi:10.3389/fnins.2021.654003)
Supplement: Supplementary file 1 [file Table_1.docx]

**Supplementary Figure 1:** An impression of the EHC measurement set-up (permission for publication was obtained).


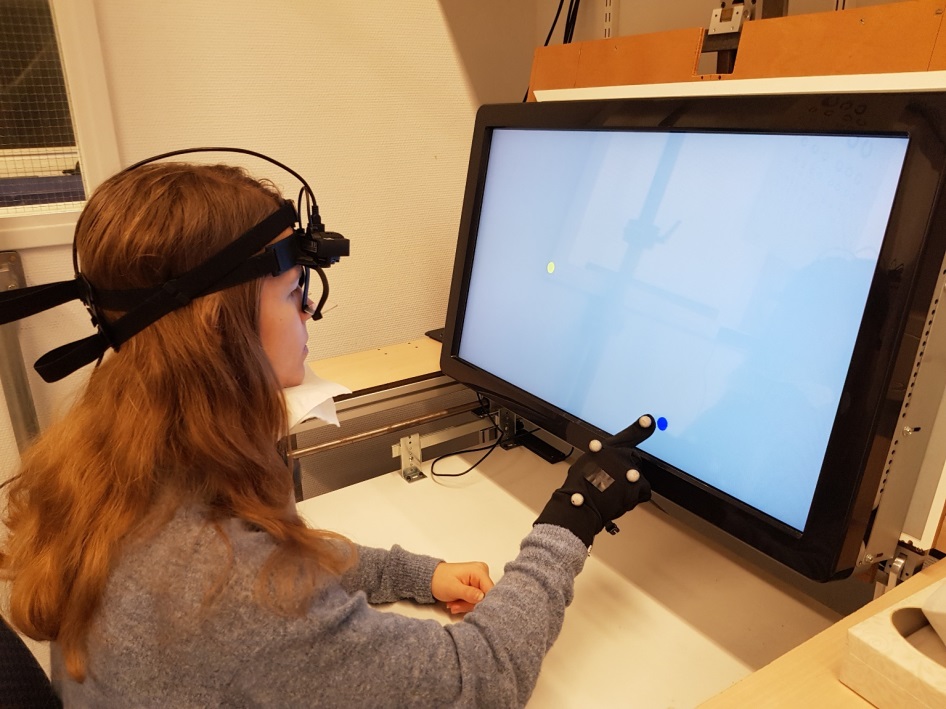


**Supplementary Figure 2:** Top panel: Schematic overview of the eye movement tasks. F: fixation before a task. PS: pro-saccade task. AS: anti-saccade task. MS: memory-saccade task (2 stages). Bottom panel: Schematic overview of the eye-hand movement tasks. F: fixation before a task. PT: pro-tapping task. AS-AT: anti-saccade anti-tapping task. AT: anti-tapping task. MT: memory-tapping task (2 stages). ST: sequential-tapping task.


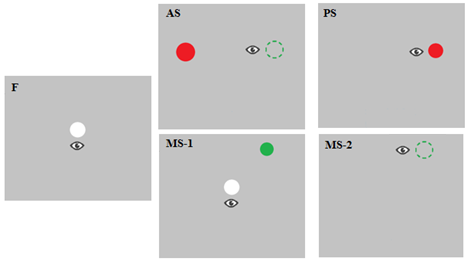


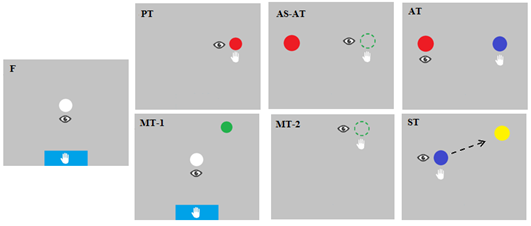


**Supplementary Figure 3:** Example of eye and hand traces. The graphs illustrate how eye latency, eye maximum velocity, hand latency, hand execution time, and hand maximum velocity are calculated.


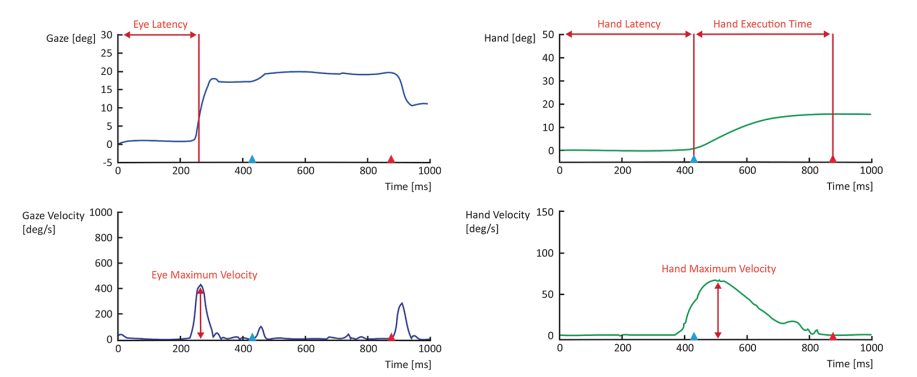

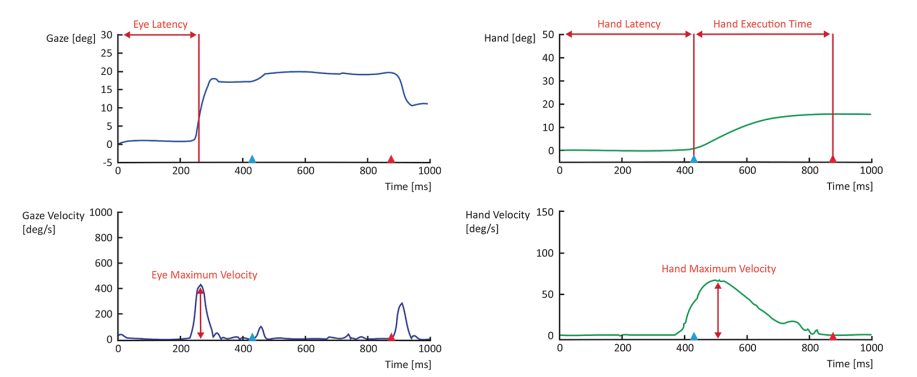


**Supplementary Table 1** Number of participants per group who completed each eye and eye/hand task.

| Task | Controls (*n* = 37) | MCI patients(*n* = 22) | AD patients(*n* = 37) |
| --- | --- | --- | --- |
| Pro-saccade task | 24 | 26 | 11 |
| Anti-saccade task | 22 | 21 | 9 |
| Memory-saccade task | 4 | 1 | 1 |
| Pro-tapping task | 36 | 37 | 22 |
| Anti-saccade anti-tapping task | 14 | 17 | 10 |
| Anti-tapping task | 15 | 14 | 6 |
| Memory-tapping task | 27 | 12 | 5 |
| Sequential tapping task | 15 | 27 | 13 |

**Supplementary Table 2** Feature selection results, ranked by IG, for the controls – MCI, controls – AD, and MCI – AD dataset views. AS: anti-saccade task. ASAT: anti-saccade anti-tapping task. AT: anti-tapping task. PS: pro-saccade task. PT: pro-tapping task. ST: sequential tapping task.

| Controls – MCI | | Controls – AD | | MCI - AD | |
| --- | --- | --- | --- | --- | --- |
| Variables | IG | Variables | IG | Variables | IG |
| Hand movement time (stimulus 1) (ST) | 0.187 | Hand latency (PT) | 0.190 | Hand latency (PT) | 0.165 |
| Hand movement time (stimulus 2) (ST) | 0.157 | Hand latency (stimulus 2) (ST) | 0.186 | Pupil latency (AS) | 0.028 |
| Eye touch interval (stimulus 1) (ST) | 0.110 | Hand movement time (stimulus 1) (ST) | 0.161 | Hand total distance (ASAT) | 0.005 |
| Eye touch interval (stimulus 2) (ST) | 0.077 | Hand movement time (PT) | 0.132 | Hand error (AT) | 0.004 |
| Hand error (ASAT) | 0.048 | Eye-hand interval (PT) | 0.126 | Hand movement time (AT) | 0.004 |
| Eye latency (ASAT) | 0.034 | Hand latency (stimulus 1) (ST) | 0.118 | Hand latency (AT) | 0.004 |
|  |  | Eye latency (AS) | 0.113 | Hand maximum velocity (ASAT) | 0.003 |
|  |  | Hand movement time (stimulus 2) (ST) | 0.108 | Hand maximum velocity (AT) | 0.002 |
|  |  | Eye touch interval (stimulus 1) (ST) | 0.097 |  |  |
|  |  | Pupil latency (AS) | 0.077 |  |  |
|  |  | Saccadic error (PS) | 0.067 |  |  |
|  |  | Anticipation (stimulus 2) (ST) | 0.016 |  |  |

**Supplementary Table 3** Descriptive statistics of the variables selected based on IG

| EHC task | Variable | Group | *N* | Mean ± standard deviation |
| --- | --- | --- | --- | --- |
| Pro-saccade | Saccadic error | Controls  MCI  AD | 23  11  26 | 3.6 ± 1.3 deg  4.5 ± 1.6 deg  5.2 ± 1.8 deg |
| Anti-saccade | Eye latency | Controls  MCI  AD | 23  3  16 | 384 ± 88 ms  319 ± 71 ms  492 ± 358 ms |
|  | Saccadic error | Controls  MCI  AD | 23  3  15 | 10.1 ± 3.7 deg  17.0 ± 4.3 deg  14.0 ± 6.2 deg |
|  | Pupil latency | Controls  MCI  AD | 19  5  13 | 347 ± 298 ms  256 ± 195 ms  1159 ± 571 ms |
| Pro-tapping | Hand latency | Controls  MCI  AD | 35  21  37 | 492 ± 74 ms  500 ± 78 ms  683 ± 310 ms |
|  | Eye-hand interval | Controls  MCI  AD | 33  21  28 | 240 ± 66 ms  251 ± 75 ms  390 ± 233 ms |
|  | Hand movement time | Controls  MCI  AD | 35  21  37 | 518 ± 147 ms  637 ± 217 ms  603 ± 163 ms |
| Sequential tapping | Hand latency (stimulus 1) | Controls  MCI  AD | 27  13  27 | 582 ± 102 ms  597 ± 95 ms  767 ± 232 ms |
|  | Hand latency (stimulus 2) | Controls  MCI  AD | 27  10  27 | 345 ± 164 ms  572 ± 255 ms  553 ± 208 ms |
|  | Hand movement time (stimulus 1) | Controls  MCI  AD | 27  13  27 | 477 ± 79 ms  729 ± 178 ms  644 ± 193 ms |
|  | Hand movement time (stimulus 2) | Controls  MCI  AD | 27  13  26 | 506 ± 100 ms  663 ± 165 ms  596 ± 152 ms |
|  | Eye touch interval (stimulus 1) | Controls  MCI  AD | 25  10  22 | 730 ± 115 ms  967 ± 178 ms  900 ± 284 ms |
|  | Anticipation (stimulus 2) | Controls  MCI  AD | 13  4  9 | *Range*: 1 – 2 trial(s)  1 trial(s)  1 trial(s) |
| Anti-saccade anti-tapping | Eye latency | Controls  MCI  AD | 14  4  4 | 617 ± 200 ms  277 ± 100 ms  351 ± 303 ms |
|  | Hand error | Controls  MCI  AD | 14  4  5 | 20.4 ± 7.7 deg  6.4 ± 1.8 deg  15.4 ± 11.1 deg |
